# Safety and efficacy of herbal extracts to restore respiratory health and improve innate immunity in COVID-19 positive patients with mild to moderate severity: A structured summary of a study protocol for a randomised controlled trial

**DOI:** 10.1186/s13063-020-04906-x

**Published:** 2020-11-23

**Authors:** Hrishikesh Rangnekar, Suresh Patankar, Kishor Suryawanshi, Pravin Soni

**Affiliations:** 1 Quest Clinicals and Ayurceuticals, Pune, India; 2 AMAI Charitable Trust, Pune, India; 3 YCM Hospital, Pune, India

**Keywords:** COVID-19, Randomised controlled trial, protocol, CAM, Ayurveda, Herbal, Herbo-mineral, Viral load

## Abstract

**Objectives:**

Primary Objective

• To assess the efficacy of herbal extracts in boosting innate immunity of patients with COVID-19 infection.

Secondary Objectives

• To assess the efficacy of herbal extracts in restoring respiratory health

• To assess the efficacy of Cap. IP in early recovery of patients and decline in viral load

• To assess the safety of herbal extracts

**Trial design:**

This is a single centre, randomized, 2-arm, parallel group, double blind, 1:1 ratio, controlled, exploratory trial with a study period of 30 days from the day of enrolment.

**Participants:**

Patients attending the COVID treatment centre at Yashwantrao Chavan Memorial Hospital, Nehrunagar, Pimpri, Pune, India were screened for their participation in the study. Patients who were known COVID-19 positive (with positive RT-PCR), eligible and willing were enrolled in the study.

**Intervention and comparator:**

The intervention in the trial has a background in ‘Ayurved’. Intervention Arm: Two capsules, Investigational Product (IP) - 1 - 400mg and Investigational Product - 2 - 450mg, containing herbal extracts (a blend of water and CO_2_ extracts) of Shunthi (*Zingiber officinale* (Ginger), Vidanga (*Embelia ribes*), Yashtimadhu (*Glycyrrhiza glabra*), Haritaki (*Terminalia chebula*), Guduchi (*Tinospora cordifolia*), Shatavari (*Asparagus racemosus*), Aamalaki (*Emblica officinalis*), Pippali (*Piper longum*) and calcined Zinc, Shankha bhasma.

Placebo Arm: Edible starch ~ 450 mg. The look and feel of IP and of Placebo boxes were very similar.

Patients are to take two capsules (one each of IP-1 and IP-2) twice a day for 15 days, and from the 16th day, one capsule of IP-2 twice a day up-to day 30. Capsules are to be administered orally with plain water.

The IP is to be taken with all other concomitant medicines prescribed by the treating physician/doctor.

The dose of each component in the IP is very safe to administer. The investigational products are registered products with the Indian Government and have been used for more than 6 months in various health conditions but not for COVID-19.

**Main outcomes:**

Primary Outcome:

Efficacy of the herbal extracts in COVID 19 positive patients (in declining viral load: time-point: 4 days and early recovery)

Secondary Outcomes:

Efficacy of the herbal extracts as an immune-modulator - TH1, TH2, Th17, IL6, NK Cells and CD markers; Immunoglobulin IGG (Serum); Immunoglobulin IGM (Serum) - at 30 days.

Efficacy of the investigational product in reducing sequela of the disease

Safety analysis (Liver Function Test and Kidney Function Test) including serious allergic reaction of: rash, itching/swelling, severe dizziness, trouble breathing.

**Randomisation:**

An alphanumeric coded set of IP/Placebo containers will be used. Participants will be automatically randomized to two groups in the ratio 1:1.

**Blinding (masking):**

Participants, caregivers and investigators were blinded.

**Numbers to be randomised (sample size):**

A total of more than 60 and up to 75 patients were to be enrolled in the study into the two groups, considering drop-outs. 72 were enrolled with 37 into the intervention group and 35 into the placebo group.

**Trial Status:**

Protocol number: CoviQuest-01

Protocol version number: 1.2

Protocol Date: 1^st^ July 2020

The recruitment period is completed for the trial. Date of 1^st^ patient enrolment was 11^th^ Aug 2020 and the last patient was enrolled on 3^rd^ of September 2020.

This is to state that it was a late submission from authors for publication of the protocol to the BMC, after enrolment in the study was over.

Last Participant’s last follow-up is scheduled on 5^th^ October 2020

**Trial registration:**

The trial was prospectively registered with the CTRI (Clinical Trial Registry of India). Registration number is CTRI/2020/07/026570. Registered on 14 July 2020

**Full protocol:**

The full protocol is attached as an additional file, accessible from the Trials website (Additional file 1). In the interest in expediting dissemination of this material, the familiar formatting has been eliminated; this Letter serves as a summary of the key elements of the full protocol.

## Supplementary Information

**Additional file 1.** Full Study Protocol.

## Data Availability

Data of the participants will be filled in by the study coordinator. Co-PI and PI will have rights to correct the data whenever needed. All the procedures will be carried out by adhering to the Good Clinical Practices (GCP). The monitor will have access to the study documents. The sponsor of the study can audit the study with prior appointment with the PI and the study coordinator.

